# Inhibitory Activities of Blasticidin S Derivatives on Aflatoxin Production by *Aspergillus Flavus*

**DOI:** 10.3390/toxins9060176

**Published:** 2017-05-26

**Authors:** Tomoya Yoshinari, Yoshiko Sugita-Konishi, Takahiro Ohnishi, Jun Terajima

**Affiliations:** 1 Division of Microbiology, National Institute of Health Sciences, 1-18-1 Kamiyoga, Setagaya-ku, Tokyo 158-8501, Japan; ohnisi@nihs.go.jp (T.O.); terajima@nihs.go.jp (J.T.); 2 Department of Food and Life Sciences, Azabu University, 1-17-71 Fuchinobe, Chuo-ku, Sagamihara, Kanagawa, Tokyo 252-5201, Japan; y-konishi@azabu-u.ac.jp

**Keywords:** aflatoxin, blasticidin S, structure–activity relationship

## Abstract

Blasticidin S (BcS) is a protein synthesis inhibitor which shows strong growth inhibitory activity against a number of microorganisms. However, BcS inhibited aflatoxin production by *Aspergillus flavus* without affecting its growth. In order to obtain information about the structure–activity relationship of BcS as an aflatoxin production inhibitor, BcS derivatives were prepared and their aflatoxin production inhibitory activities were evaluated. Among five derivatives, blasticidin S carboxymethyl ester, deaminohydroxyblasticidin S, and pyrimidinoblasticidin S showed inhibitory activity, while the others did not. The IC_50_ value for aflatoxin production of the carboxymethyl ester derivative was one-fifth of that of BcS although their antimicrobial activities were almost the same. These results indicate that the inhibitory activity of BcS against aflatoxin production was enhanced by esterification of its carboxyl group and that the carboxymethyl ester derivative might be more suitable for practical use than BcS because of the specificity of the carboxymethyl ester derivative, which inhibited aflatoxin production more than BcS.

## 1. Introduction

**Figure 1:**
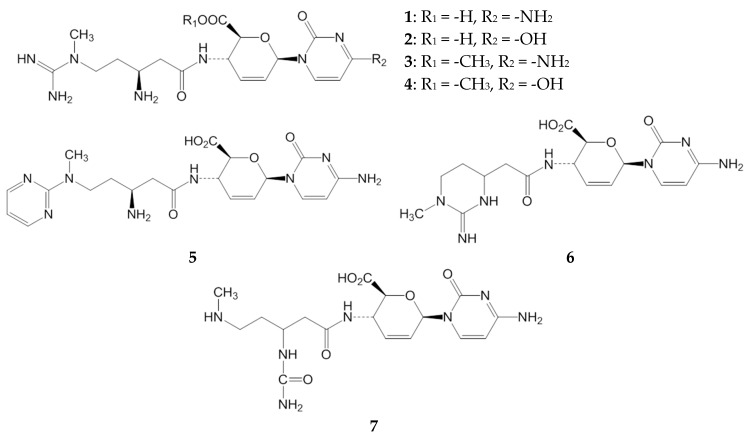
Structures of BcS and its derivatives.

Aflatoxins are a group of secondary metabolites produced by some *Aspergillus* species, including *A. flavus* and *A. parasiticus*, and are detected in many kinds of food and feed commodities such as corn, tree nuts, pulses, dried fruit, and spices. Because they are potent mutagens and are liver carcinogens in humans and a wide range of animal species, aflatoxin contamination in crops causes huge economic losses and may constitute a serious health threat [1,2,3,4]. Some attempts have been made to regulate aflatoxin contamination in food, including the use of atoxigenic strains of *Aspergilli*, but no successful methods have yet been established [5,6]. A practical use of aflatoxin production inhibitors is expected to be an effective method to prevent aflatoxin contamination in agricultural products. Many kinds of aflatoxin production inhibitors including blasticidin S (BcS, **1** in Figure 1) have already been reported, and most of them are derived from plant constituents, microbial metabolites, or synthetic compounds [7,8].

BcS is a *Streptomyces griseochromogenes* metabolite that is known to block protein synthesis in both bacteria and eukaryotes by inhibiting peptidyl transfer on their ribosomes [9,10]. BcS shows strong growth inhibitory activity against a number of microorganisms such as *Piricularia oryzae*, *Escherichia coli*, and *Saccharomyces cerevisiae* [11,12]. However, some *Aspergillus* species, including *A. flavus*, *A. fumigatus*, and *A. terreus*, are highly resistant to BcS because they can metabolize BcS into a non-toxic metabolite, deaminohydroxyblasticidin S (DahBcS, **2** in Figure 1) [12,13,14]. We previously reported the inhibitory activities of BcS and DahBcS on aflatoxin production by *A. flavus* [15]. These compounds did not affect the growth of *A. flavu*s and specifically inhibited its aflatoxin production. A compound that specifically decreases aflatoxin production but does not inhibit the growth of the aflatoxin producer and other organisms is desirable in practical applications because the influence on organisms in the environment should be kept to a bare minimum. The specificity of DahBcS on the inhibition of aflatoxin production is higher than that of BcS because of the low toxicity of DahBcS against microorganisms and mammalian cells [12], but the inhibitory activity of DahBcS on aflatoxin production is much lower than that of BcS [15].

A study on the structure–activity relationship of BcS would be useful to develop effective aflatoxin production inhibitors. Previously, the structure–activity relationship of BcS as a peptidyl transferase inhibitor was assessed [16]. The effects of BcS and its derivatives on the synthesis of peptide bonds in vitro were tested, and the inhibition constants of BcS, blasticidin S carboxymethyl ester (MeBcS, **3** in Figure 1), *N*-acetylblasticidin S, and DahBcS were 0.63, 2.5, 40, and 250, respectively. In another study, the effects of BcS and its related compounds on polypeptide synthesis in the *E. coli* subcellular system were evaluated [17]. The inhibitory activity of DahBcS was 1000-fold lower than that of BcS, and cytomycin (**6** in Figure 1), an alkaline hydrolysate of BcS, completely failed to inhibit polypeptide synthesis. These results suggest that the cytosine moiety and the free amino group at C-13 in BcS are important for peptidyl transferase inhibition. On the other hand, there is little information about the structure–activity relationship of BcS as an aflatoxin production inhibitor. In this study, we prepared several BcS derivatives, evaluated their inhibitory activity against aflatoxin production by *A. flavus* and growth inhibitory activity against some microorganisms, and the specificity on the inhibition of aflatoxin production was compared.

## 2. Results and Discussion

### 2.1. Inhibitory Activities of BcS Derivatives on Aflatoxin Production

**Figure 2:**
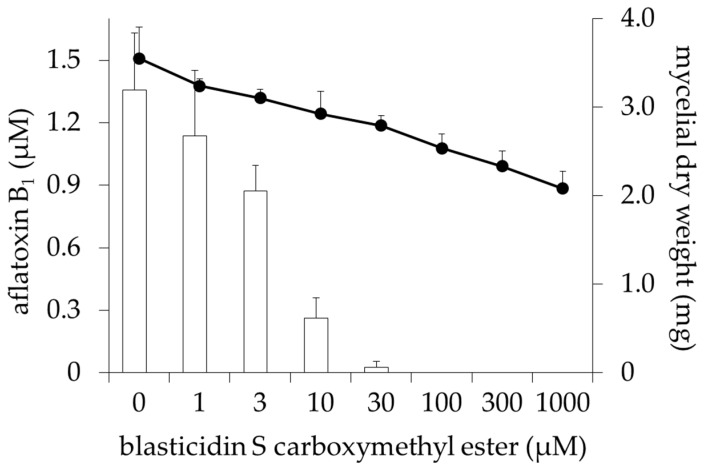
Effects of blasticidin S carboxymethyl ester (MeBcS) on aflatoxin production and fungal growth. *A*. *flavus* IMF 47798 was cultured in PD liquid medium with or without MeBcS at 30 °C for four days. The amount of aflatoxin B_1_ (white bars) in the culture filtrate was determined by LC-MS/MS. The mycelial cake was lyophilized for 12 h and weighed (●). Data are presented as the mean ± SD (*n* = 6).

**Figure 3:**
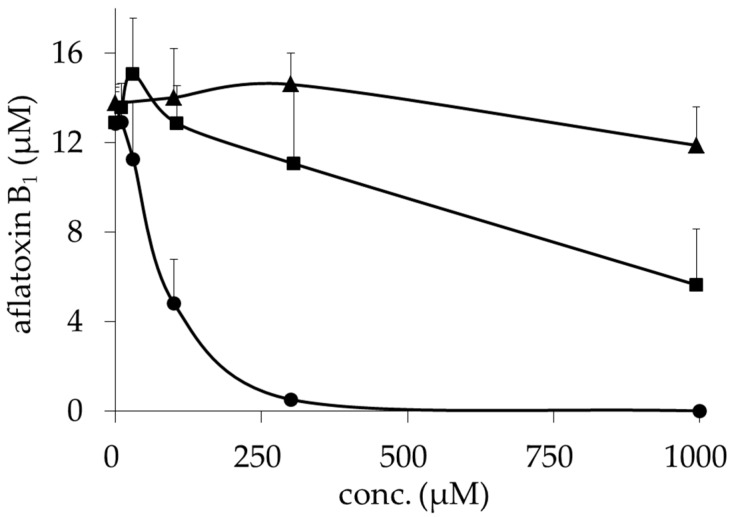
Effects of blasticidin S (■), blasticidin S carboxymethyl ester (●) and deaminohydroxyblasticidin S (▲) on aflatoxin production by *A*. *flavus* IMF 47798 in a YES liquid medium. The amount of aflatoxin B_1_ in the culture filtrate was quantitated by LC-MS/MS. Data are presented as the mean ± SD (*n* = 6).

Pyrimidinoblasticidin S (PyBcS, **5** in Figure 1) was prepared by modifying the guanidine group of BcS. Liberation of one mole of ammonia from BcS under an alkaline condition gave cytomycin. Cytomycin was further degraded into **7** under an alkaline condition. MeBcS was obtained by converting the carboxyl group of BcS into methyl ester. DahBcS was prepared by microbial transformation of BcS. ^1^H NMR and ^13^C NMR data of the prepared derivatives are shown in Supplementary Materials (Table S1). *A. flavus* IMF 47798 strain was cultured in a potato dextrose (PD) broth medium with or without BcS derivatives and their inhibitory activities on aflatoxin production were evaluated (Table 1). BcS and DahBcS inhibited aflatoxin production, as shown in our previous study [15], and their IC_50_ values were 27 and 110 μM, respectively (Table 1). Cytomycin and **7** did not inhibit aflatoxin production and their IC_50_ values exceeded 1000 μM. MeBcS inhibited aflatoxin production in a dose-dependent manner with an IC_50_ value of 5 μM, which was about one-fifth of the IC_50_ value of BcS (Figure 2). MeBcS decreased the mycelial weight of *A. flavus* significantly, but fungal growth was observed even at 1000 μM. PyBcS also inhibited aflatoxin production and the IC_50_ value of PyBcS was 20 times higher than that of BcS. Next, the inhibitory activities of BcS, DahBcS, and MeBcS against aflatoxin production by *A. flavus* IMF 47798 strain in a broth medium containing yeast extract and sucrose (YES medium) were examined. The result is shown in Figure 3. The amount of AFB_1_ produced by this strain in YES medium was about 10 times greater than that in PD broth medium. BcS partially inhibited aflatoxin production by *A. flavus* and the IC_50_ value was 890 μM, while DahBcS could not inhibit aflatoxin production at all. MeBcS inhibited aflatoxin production dose-dependently (IC_50_ value = 80 μM) and AFB_1_ was not detected in the culture containing 1000 μM MeBcS. BcS, DahBcS, and MeBcS did not affect the growth of *A. flavus*, even at 1000 μM.

Since the IC_50_ value of DahBcS was higher than that of BcS, the presence of the amino group in the cytosine moiety is needed for full inhibitory activity. Although PyBcS, cytomycin, and **7** have a cytosine moiety, their inhibitory activities were weaker than those of DahBcS. This means that not only a cytosine moiety but also a β-amino group and a guanidine group are important for this activity. The carboxy group in the sugar portion is not critical for activity because MeBcS, a BcS carboxymethyl ester derivative, inhibited aflatoxin production by *A. flavus* more strongly than BcS. In our previous study, BcS repressed the mRNA levels of *pksA*, *omtA*, and *aflR* [15]. *PksA* and *omtA* encode enzyme proteins involved in aflatoxin biosynthesis, and *aflR* encodes a transcriptional factor which regulates the transcription of aflatoxin biosynthesis enzymes. MeBcS also decreased the mRNA levels of these genes (Figure S1). This means that MeBcS as well as BcS might affect a regulatory system that controls aflatoxin production.

### 2.2. Antimicrobial Activity of BcS Derivatives

In order to assess the specificity of BcS derivatives on the inhibition of aflatoxin production, their growth inhibitory activities against *Aspergillus niger*, *Saccharomyces cerevisiae*, and *Escherichia coli* were evaluated. *A*. *niger* was cultured statically in PD broth medium and mycelial weight was measured after incubation. *S. cerevisiae* and *E. coli* were cultured under shaking conditions and optical density at 595 nm was measured after incubation. The MIC was defined as the concentration at which the growth of a microorganism was completely inhibited (Table 1). BcS and MeBcS inhibited the growth of *A. niger*, *S cerevisiae*, and *E. coli*. The MICs of *A. niger*, *S. cerevisiae*, and *E. coli* to BcS were 300, 3, and 500 µM, respectively, while those to MeBcS were 300, 10, and 300 µM, respectively. Other derivatives did not inhibit the growth of these three microorganisms.

The levels of antimicrobial activitiy of BcS and MeBcS were similar, but the inhibitory activity of MeBcS on aflatoxin production was stronger than that of BcS. DahBcS showed no antimicrobial activity although it inhibited aflatoxin production by *A. flavus* in PD broth medium. These results indicate that MeBcS and DahBcS are more highly specific inhibitors of aflatoxin production than BcS. Furthermore, only MeBcS could inhibit aflatoxin production by *A. flavus* in YES medium. Therefore, MeBcS was the best inhibitor in this study because it could inhibit aflatoxin production specifically under a highly aflatoxin-producing condition.

### 2.3. Determination of the Deaminated Metabolites of Blasticidin S and Its Methyl Ester

**Figure 4:**
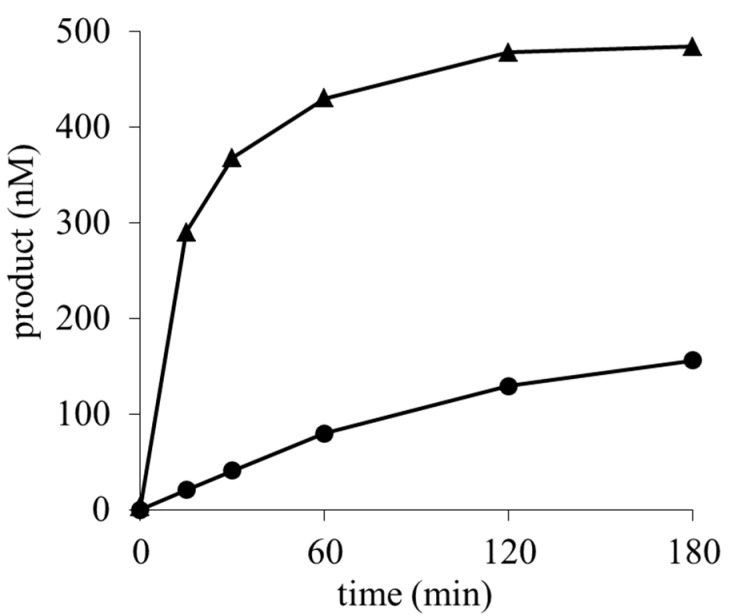
In vitro conversion experiments of blasticidin S and its carboxymethyl ester. Cell free extracts from *A. flavus* IMF 47798 were incubated with blasticidin S or its carboxymethyl ester, and the production of deaminohydroxyblasticidin S (▲) or its carboxymethyl ester (●) was quantified.

BcS and MeBcS inhibited both aflatoxin production by *A. flavus* and the growth of the three tested microorganisms. In contrast, the BcS derivatives, which showed no aflatoxin inhibitory activity, also did not inhibit the growth of these microorganisms. Therefore, the mode of action of the inhibitory activity of BcS on aflatoxin production might be caused by its inhibitory activity on protein synthesis. A previous study showed that the in vitro inhibitory activities of BcS and MeBcS on protein synthesis were equivalent [16]. In our study, the antimicrobial activity of BcS was comparable to that of MeBcS. However, the IC_50_ value for aflatoxin production of MeBcS was one-fifth of that of BcS. In order to clarify the reason why the inhibitory activity of MeBcS on aflatoxin production was stronger than that of BcS, even though the antifungal activity of BcS was similar to that of MeBcS, the metabolites of BcS and MeBcS in *A. flavus* were determined. As mentioned in the introduction, a toxic compound, BcS, is transformed into a non-toxic metabolite, DahBcS, by BcS deaminase in *A. flavus*, and it has been reported that the activity of BcS deaminase in *A. flavus* was enhanced when cultured in the presence of BcS [15]. In order to know whether MeBcS influences the transcription of the enzyme, *A. flavus* IMF 47798 strain was cultured with BcS or MeBcS, and the mRNA level of BcS deaminase was analyzed by quantitative PCR. As shown in Figure S2, both BcS and MeBcS increased the transcription of the enzyme. This means that MeBcS also enhances the deaminase activity in *A. flavus* and may be metabolized into a non-toxic deaminated compound in vivo. Next, it was expected that the metabolic rates of BcS and MeBcS in *A. flavus* would be different. BcS or MeBcS was incubated with a cell-free extract from *A. flavus* and the deaminated metabolites were quantitated (Figure 4). DahBcS was produced from BcS over time, and 500 nM BcS was metabolized into 480 nM DahBcS by 120 min. MeBcS was also deaminated by BcS deaminase. Deaminohydroxyblasticidin S methyl ester (DahMeBcS, **4** in Figure 1) was detected in the reaction mixture, and 500 nM MeBcS was metabolized into 130 nM DahMeBcS by 120 min. This result indicates that MeBcS is transformed into a deaminated metabolite less efficiently and can remain in the mycelia longer than BcS. The strong inhibitory activity of MeBcS on aflatoxin production might be considered to be caused by its high resistance to the metabolic enzyme for BcS. In order to verify this hypothesis, further studies including analysis of the protein level of BcS deaminase and quantitation of the metabolite of MeBcS are needed, and these experiments are now in progress.

## 3. Conclusions

In this study, a BcS derivative that shows specific and strong inhibitory activity against aflatoxin production has been found. The inhibitory activity of BcS on aflatoxin production was enhanced by esterification of its carboxyl group and the IC_50_ value for aflatoxin production of MeBcS, a carboxymethyl ester derivative, was one-fifth of that of BcS although their antimicrobial activities were at comparable levels. MeBcS was resistant to a metabolic enzyme for BcS and this might have brought about the strong inhibitory activity of the derivative on aflatoxin production. MeBcS is expected to be more suitable for practical use than BcS because the specificity of MeBcS to inhibit aflatoxin production is higher than that of BcS.

## 4. Materials and Methods

### 4.1. Strains, Growth Media, and Chemicals

*Aspergillus flavus* strain IMF 47798 was used as a mycotoxin producer. *Aspergillus niger* strain KJ16 was used for the antifungal assay. These strains were maintained on potato dextrose agar (Difco, Sparks, MD, USA) and subcultured monthly. A spore suspension was prepared from a two-week-old culture, at a concentration of 2.5 × 10^3^ c.f.u./µL and used as inoculum for this study. Potato dextrose (PD) (Becton, Dickinson and Company, Franklin Lakes, NJ, USA) broth medium and yeast extract–sucrose (YES) broth medium (2% yeast extract, 0.2% sucrose) were used for the production of aflatoxin. All cultures were incubated at 30 °C. *Escherichia coli* strain TOP10 (Thermo Fisher Scientific Inc., Waltham, MA, USA) and *Saccharomyces cerevisiae* strain INVSc1 (Thermo Fisher Scientific Inc.) were used for the antimicrobial assay. These strains were preserved in glycerol stocks at −80 °C. Luria-Bertani (LB) liquid medium (Difco) and synthetic dextrose (SD) liquid medium (0.67% yeast nitrogen base without amino acids, 0.5% casamino acids, 0.2% glucose, 0.002% uracil and 0.002% tryptophan) were used to culture *E. coli* and *S. cerevisiae*, respectively. Blasticidin S HCl (Kaken Pharmaceutical, Tokyo, Japan) was dissolved in distilled water and was stored at −20 °C. *Aspergillus terreus* strain TSY 578 was cultured in YE liquid medium (2.5% yeast extract) containing 300 µM blasticidin S, and deaminohydroxyblasticidin S was purified from the culture broth according to the methods in our previous work [15].

### 4.2. Preparation of BcS Derivatives

#### 4.2.1. Carboxymethyl Esters of Blasticidin S (3) and Deaminohydroxyblasticidin S (4)

Esterification of BcS was performed according to the method of Otake et al. [18]. BcS HCl (100 mg) was dissolved in 3 mL of super-dehydrated methanol (Wako, Osaka, Japan) containing 3% HCl and the reaction mixture was incubated at 50 °C for 5 h. The solution was neutralized by ammonium hydroxide and was evaporated to dryness. The residue was resolved in 1 mL of water and subjected to HPLC system. From 100 mg of BcS, 76 mg of MeBcS (HRESI-TOF/MS *m*/*z* 437.2262 [M + H]^+^; calcd for C_18_H_29_N_8_O_5_, 437.2261) was obtained. DahBcS (2) was prepared by microbial transformation of BcS according to our previous method [15]. Esterification of DahBcS was performed in the same way and 81 mg of DahMeBcS (HRESI-TOF/MS m/z 438.2098 [M + H]^+^; calcd for C_18_H_28_N_7_O_6_, 438.2101) was obtained from 100 mg of DahBcS.

#### 4.2.2. Pyrimidinoblasticidin S (5)

PyBcS was prepared according to the method of Yamaguchi et al. [19]. BcS HCl (100 mg) was dissolved in 2 mL of 12N HCl (Wako), and 0.22 mL of 1,1,3,3-tetraethoxypropane (Nacalai Tesque, Kyoto, Japan) was gradually added to the solution with stirring. After overnight incubation at room temperature, the solvent was evaporated to dryness. The residue was resolved in 1 mL of water and subjected to an HPLC system. From 100 mg of BcS, 58 mg of PyBcS (HRESI-TOF/MS *m*/*z* 459.2107 [M + H]^+^; calcd for C_20_H_26_N_9_O_5_, 459.2104) was obtained.

#### 4.2.3. Cytomycin (6) and (7)

Cytomycin and 7 were simultaneously produced by mild alkaline hydrolysis of BcS [20]. BcS HCl (100 mg) was dissolved in 5 mL of 0.1N NaOH and the solution was incubated at room temperature for seven days. The solvent was subjected to HPLC. From 100 mg of BcS, 60 mg of cytomycin (HRESI-TOF/MS *m*/*z* 406.1844 [M + H]^+^; calcd for C_17_H_24_N_7_O_5_, 406.1839) and 24 mg of 7 (HRESI-TOF/MS *m*/*z* 424.1941 [M + H]^+^; calcd for C_17_H_26_N_7_O_6_, 424.1945) were obtained.

#### 4.2.4. Chromatographic Condition for Purification of BcS Derivatives

The LC system consisted of a Prominence LC-20A series (Shimadzu Corp., Kyoto, Japan). Each of the BcS derivatives was purified using the following LC conditions: mobile phase, water containing 0.1% trifluoroacetic acid/acetonitrile; linear gradient of 5–21% acetonitrile in 12 min, hold at 21% acetonitrile for 3 min, followed by equilibration in 5% acetonitrile for 15 min before the next injection. The flow rate was 4 mL/min, and the column used was a 250 × 10 mm i.d., 5 μm, Inertsil ODS-3 (GL Sciences Inc., Tokyo, Japan). UV detection was set at 260 nm. The column oven was held at 40 °C. The retention times of MeBcS, DahMeBcS, PyBcS, cytomycin, and 7, were 7.8, 8.9, 9.3, 8.9, and 6.2 min, respectively. The derivatives were obtained as trifluoroacetate salts. In order to remove trifluoroacetate, each compound was subjected to the LC system [column, 250 × 10 mm i.d., 5 μm, InertSustain AQ-C18 (GL Sciences Inc.); isocratic elution of 5% acetonitrile in water; flow rate, 4.0 mL/min; detection at 260 nm]. The stock solution (50 mM) was prepared by dissolving each derivative in water.

### 4.3. Analysis of Aflatoxin B_1_

Aflatoxin B_1_ for an analytical standard was purchased from Sigma-Aldrich, St. Louis, MO, USA. It was dissolved in acetonitrile and was stored at −20 °C. A spore suspension of *A. flavus* IMF 47798 (10 μL) was added to PD medium (2 mL) with or without BcS derivatives in a well of a 12-well microplate and incubated statically for four days. The culture broth was separated into mycelium and culture by filtration. The culture filtrate was diluted 40 times with water, and aflatoxin B_1_ in the solution was quantitated by a 3200 Q TRAP LC-MS/MS system (AB Sciex, Foster City, CA, USA), equipped with an ESI source and a Prominence LC-20A series HPLC system. When YES broth medium was used for culture, the incubation was performed for five days and the culture filtrate was diluted 400 times with water before LC-MS/MS analysis. The column used was a 150 × 2.1 mm i.d., 3 μm, InertSustain C18 (GL Sciences Inc.). Chromatographic separation was achieved at 40 °C, using a gradient elution of 40–90% methanol in water containing 2 mM ammonium acetate from 0 to 8 min and then an isocratic elution of 90% methanol in water containing 2 mM ammonium acetate from 8 to 9 min at a flow rate of 0.2 mL/min. The injection volume was 5 μL. The retention time was 7.9 min. The ESI source was operated at 500 °C in the positive ionization mode. Other MS parameters were as follows: curtain gas at 10 psi, nebulizer gas (GS1) at 60 psi, turbo heater gas (GS2) at 50 psi, collision-activated dissociation gas at 8 (arbitrary units), multiple reaction monitoring, dwell time of 250 ms, and a 5 ms pause between mass ranges. The following multiple reaction monitoring transition was used: aflatoxin B_1_, 313 [M + H]^+^ to 241 (collision energy, 41 eV).

### 4.4. Analysis of Antifungal Activity of BcS and Its Derivatives

A spore suspension of *A. niger* KJ16 (5 μL) was added to PD medium (2 mL) in each well of a 12-well microplate. BcS or its derivative was added to the medium and incubated statically for four days. The culture broth was separated into mycelium and culture filtrate by filtration. The mycelium was collected into a 5 mL microtube and lyophilized for 12 h. Mycelial weight was calculated by subtracting the weight of a 5 mL microtube without the mycelium from the total weight.

### 4.5. Analysis of Antimicrobial Activity of BcS and Its Derivatives

An overnight culture of *E. coli* TOP10 was diluted 500 times with LB liquid medium. In *S. cerevisiae* INVSc1 strain, an overnight culture was diluted 50 times with SD liquid medium. Diluted suspension (100 μL) was dispensed into each well of a 96-well microplate. BcS or its derivative was added to the well at concentrations between 0.1 and 1000 μM. *E. coli* was cultured for 12 h at 37 °C while shaking at 1000 rpm. *S. cerevisiae* was cultured for 24 h at 30 °C while shaking at 1000 rpm. Growth was monitored by measuring the optical density at 595 nm by an iMark absorbance microplate reader (Bio-Rad, Hercules, CA, USA).

### 4.6. Assay of BcS Deaminase Activity In Vitro

A spore suspension of *A. flavus* IMF 47798 (5 µL) was added to 2 mL of YE liquid medium in a well of a 12-well microplate and incubated statically for 40 h at 30 °C. BcS (300 µM) was added to the medium and the culture plate was incubated for another 8 h. The mycelium was collected by filtration. After a washing with 10 mL of distilled water, total proteins of the mycelium were extracted using CelLytic Y Yeast Cell Lysis/Extraction Reagent (Sigma-Aldrich, St. Louis, MO, USA.) with a protease inhibitor cocktail for use with fungal and yeast extracts (Sigma-Aldrich, St. Louis, MO, USA) and 10 mM dithiothreitol. One milliliter of the reagent was used for the mycelia collected from three wells of a 12-well plate. For enzyme assay, BcS (final conc. = 0.5 mM) was incubated with 60 µL of protein extracts in phosphate-buffered saline (total volume of 300 µL) in a 30 °C water bath. The reaction was stopped by adding 600 µL of acetonitrile. After diluting 1000 times with water, the mixture was centrifuged (12,000 *g*, 10 min). The deaminated compounds in the supernatant were quantitated by a 3200 Q TRAP LC-MS/MS system (AB Sciex), equipped with an ESI source and a Prominence LC-20A series HPLC system. The column used was a 150 × 2.1 mm i.d., 3 μm, Inertsil ODS-4 (GL Sciences Inc.). Chromatographic separation was achieved at 40 °C, using a gradient elution of 0–80% acetonitrile in water containing 0.1% formic acid from 0 to 6 min and then an isocratic elution of 80% acetonitrile in water containing 0.1% formic acid from 6 to 8 min at a flow rate of 0.2 mL/min. The injection volume was 10 μL. The retention times of DahBcS and DahMeBcS were 5.9 and 6.0 min, respectively. The ESI source was operated at 600 °C in the positive ionization mode. Other MS parameters were as follows: curtain gas at 10 psi, nebulizer gas (GS1) at 50 psi, turbo heater gas (GS2) at 40 psi, collision-activated dissociation gas at 3 (arbitrary units), multiple reaction monitoring, dwell time of 250 ms, and a 5 ms pause between mass ranges. The following multiple reaction monitoring transition was used: DahBcS, 424 [M + H]^+^ to 154 (collision energy, 45 eV) and DahMeBcS, 438 [M + H]^+^ to 138 (collision energy, 49 eV).

## Figures and Tables

**Table 1:** Inhibitory activities of BcS derivatives against aflatoxin production by *A. flavus* and the growth of microorganisms.

Compound	Aflatoxin Production IC_50_ (µM)	Growth (Minimum Inhibitory Concentration (µM))			
	*A. flavus*	*A. flavus*	*A. niger*	*S. cerevisiae*	*E. coli*
BcS (**1**)	27 ^a^	>1000 ^a^	300 ^a^	3	500
DahBcS (**2**)	110 ^a^	>1000 ^a^	>1000 ^a^	>1000	>1000
MeBcS (**3**)	5	>1000	300	10	300
PyBcS (**5**)	610	>1000	>1000	>1000	>1000
cytomycin (**6**)	>1000	>1000	>1000	>1000	>1000
**7**	>1000	>1000	>1000	>1000	>1000

^a^ Inhibitory activities of BcS and DahBcS against aflatoxin production by *A. flavus* and the growth of *A. favus* and *A. niger* have been published in my previous work [15]. They are remeasured in this study.

## Supplementary Materials

The following are available online at www.mdpi.com/2072-6651/9/6/176/s1, Table S1: NMR spectroscopic data for blasticidin S derivatives, Figure S1: Effect of blasticidin S carboxymethyl ester on the transcription of genes encoding proteins involved in aflatoxin biosynthesis, Figure S2: Effect of blasticidin S carboxymethyl ester on the transcription of gene encoding proteins involved in aflatoxin biosynthesis.
