# Supplementary material for: Inhibitory Activities of Blasticidin S Derivatives on Aflatoxin Production by Aspergillus Flavus
Source: Toxins (Basel). 2017 May 26;9(6):176. doi: 10.3390/toxins9060176 (PMC5488026; doi:10.3390/toxins9060176)
Supplement: Supplementary file 1 [file toxins-09-00176-s001.pdf]

Supplementary Materials: Inhibitory Activities of Blasticidin S Derivatives on Aflatoxin Production by *Aspergillus Flavus*

Tomoya Yoshinari, Yoshiko Sugita-Konishi, Takahiro Ohnishi and Jun Terajima

Table S1. NMR spectroscopic data for blasticidin S derivatives.

position	MeBcS (**3**)		DahMeBcS (**4**)		PyBcS (**5**)		cytomycin (**6**)		**7**	
	δ_C[a]	δ_H[a] (*J* in Hz)	δ_C[a]	δ_H[a] (*J* in Hz)	δ_C[b]	δ_H[b] (*J* in Hz)	δ_C[a]	δ_H[a] (*J* in Hz)	δ_C[a]	δ_H[a] (*J* in Hz)
1	148.8		151.8		148.6		148.7		148.6	
2	159.6		166.2		159.5		159.6		159.5	
3	95.6	6.24, d (7.8)	102.7	5.91, d (8.2)	95.6	6.22, d (7.8)	95.5	6.23, d (7.8)	95.5	6.24, d (7.9)
4	145.4	7.82, d (7.8)	142.7	7.64, d (8.2)	145.5	7.81, d (7.8)	145.5	7.84, d (7.8)	145.5	7.83, d (7.9)
5	79.4	6.57, s, br	78.8	6.54, s, br	79.3	6.55, s, br	79.4	6.54, s, br	79.2	6.57, s, br
6	124.6	6.26, dt (10.5, 2.5)	125.3	6.25, dt (10.4, 2.4)	124.6	6.24, dt (10.5, 2.3)	124.5	6.22, dt (10.5, 2.3)	124.2	6.25, dt (10.0, 2.4)
7	133.1	5.96, dt (10.5, 2.1)	132.4	5.99, dt (10.4, 2.0)	133.5	5.94, dt (10.5, 2.1)	134.0	5.90, dt (10.5, 2.1)	133.5	5.94, dt (10.0, 2.1)
8	44.5	4.83, ddd (8.2, 2.5, 2.1)	44.6	4.85, ddd (8.2, 2.4, 2.0)	44.5	4.83, ddd (8.2, 2.3, 2.1)	44.9	4.82, ddd (8.7, 2.3, 2.1)	44.5	4.82, ddd (8.2, 2.4, 2.1)
9	75.1	4.55, d (8.2)	75.0	4.55, d (8.2)	75.2	4.43, d (8.2)	76.3	4.28, d (8.7)	75.1	4.47, d (8.2)
10	171.3		171.2		172.1		173.3		172.7	
11	170.3		170.4		171.4		172.3		171.9	
12	36.5	2.74, m	36.5	2.77, m	36.7	2.77, m	36.5	2.52, m	41.2	2.52, m
13	46.4	3.68, m	46.4	3.70, m	46.3	3.62, m	45.7	3.84, m	45.2	4.05, m
14	29.3	2.06, m	29.2	2.09, m	29.4	2.10, m	25.4	2.12, m	31.0	1.96, m
								1.86, m		1.85, m
15	46.7	3.49, m	46.6	3.52, m	46.7	3.94, m	46.0	3.43, m	46.1	3.10, m
16	35.9	3.05, s	35.8	3.08, s	35.4	3.26, s	40.9	3.02, s	32.8	2.73, s
17	156.7		156.6		153.9		153.5		160.8	
18	-		-		157.7	8.58, s, br	-		-	
19	-		-		109.4	7.04, t (5.5)	-		-	
20	-		-		157.7	8.58, s, br	-		-	
$COO\underline{C}H_3$	53.5	3.76, s	53.4	3.78, s	-		-		-	

[a] 125 MHz (δ_C) and 500 MHz (δ_H) in D_2O. [b] 125 MHz (δ_C) and 500 MHz (δ_H) in DMSO-d6.

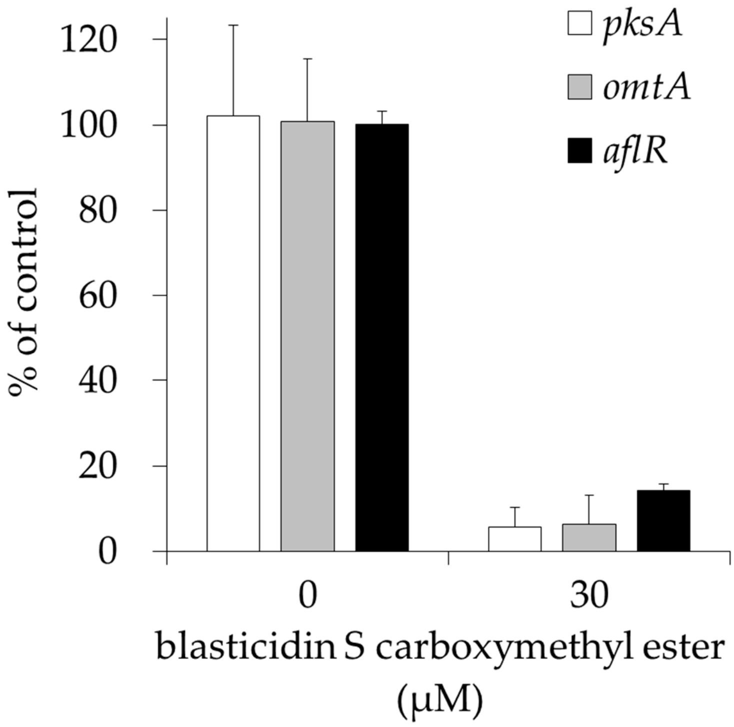

Figure S1. Effect of blasticidin S carboxymethyl ester on the transcription of genes encoding proteins involved in aflatoxin biosynthesis. The mRNA levels of the three genes were analyzed by quantitative PCR according to the method in our previous work [1]. Data are presented as the mean ± SD (n = 6).

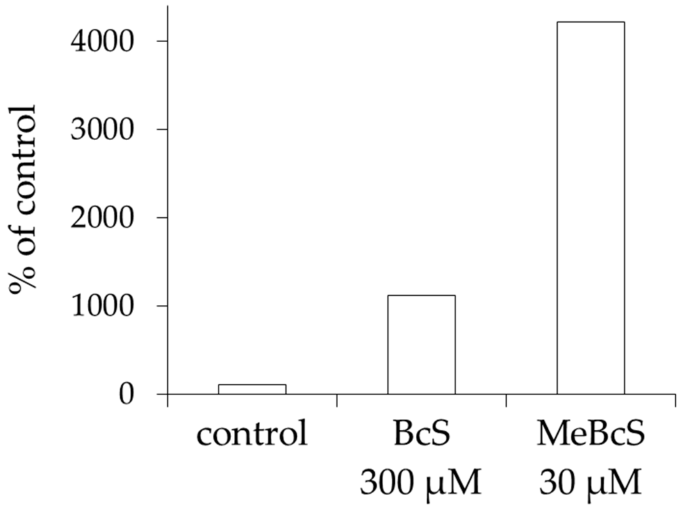

Figure S2. Effect of blasticidin S (BcS) and its carboxymethyl ester (MeBcS) on the transcription of blasticidin S deaminase. The mRNA level was analyzed by quantitative PCR according to the method in our previous work [1]. The primers used were 5′-GGCAGGTTTTGTGGGATTTG-3′ and 5′-CCCCTCCTCCCCTAAAACAAT-3′. Data are presented as the mean (n = 2).

References

1. Yoshinari, T.; Sakuda, S; Watanabe, M.; Kamata, Y.; Ohnishi, T.; Sugita-Konishi, Y. New metabolic pathway for converting blasticidin S in *Aspergillus flavus* and inhibitory activity of aflatoxin production by blasticidin S metabolites. *J. Agric. Food Chem.* **2013**, *61*, 7925–7931.
